# Effects of fertilization on drought responses in saplings of three European trees species

**DOI:** 10.1093/treephys/tpag089

**Published:** 2026-06-29

**Authors:** Feng Feng, Adriano Losso, Barbara Beikircher, Andrea Ganthaler, Melvin Tyree, Stefan Mayr

**Affiliations:** Department of Botany, University of Innsbruck, Sternwartestraße 15, Innsbruck 6020, Austria; Department of Botany, University of Innsbruck, Sternwartestraße 15, Innsbruck 6020, Austria; Department of Botany, University of Innsbruck, Sternwartestraße 15, Innsbruck 6020, Austria; Department of Botany, University of Innsbruck, Sternwartestraße 15, Innsbruck 6020, Austria; Plattsburgh 12901, NY, USA; Department of Botany, University of Innsbruck, Sternwartestraße 15, Innsbruck 6020, Austria

**Keywords:** biomass, gas exchange, hydraulics, nutrient, water stress, xylem embolism resistance

## Abstract

Nutrient and water supply are decisive limiting factors for trees and their productivity. Yet, the consequences of fertilization for drought responses, especially during the sensitive nursery stage, remain poorly understood. In this study, we exposed potted saplings of *Pinus sylvestris* L., *Fagus sylvatica* L. and *Quercus robur* L. to two levels of fertilization and three water regimes. The effects of fertilization, drought and their interaction on gas exchange, hydraulic traits and growth were examined. Fertilization generally promoted gas exchange, hydraulic conductance and biomass accumulation but responses were species-specific and did not always persist under and after drought. While fertilized *Q. robur* maintained or even increased its biomass and physiological performance under drought, the benefits of fertilization for *P. sylvestris* and *F. sylvatica* diminished under increasing drought intensity. No consistent changes in drought resistance traits (e.g., turgor loss point, embolism resistance) were observed across species in response to fertilization, which may indicate limited plasticity or acclimation potential of saplings. Our findings highlight the species-specific fertilization effects on drought responses and post-drought performance of saplings, emphasizing the need to consider both factors in silvicultural practices under future climate scenarios.

## Introduction

The frequency and intensity of drought and heat extremes have increased in recent decades and are projected to continue rising (IPCC 2021). Increasing drought stress is known to threaten forest stability and tree health, with cascading consequences for ecosystem services such as water regulation, biodiversity conservation, carbon sequestration and timber production (Glatthorn et al. 2021, Hartmann et al. 2022). Also in Central Europe, widespread tree mortality has recently been attributed to intensifying drought conditions (e.g., Schuldt et al. 2020, Senf et al. 2020, Knutzen et al. 2025), affecting tree water relations.

Water relations are a central component of tree physiology, encompassing water uptake by roots, internal storage, transport through the xylem and regulation of transpiration via stomata. Under drought conditions, these interconnected processes become increasingly strained, affecting the tree’s ability to maintain water balance and physiological function. A key mechanism underlying drought-induced tree mortality is hydraulic failure, which is primarily caused by xylem embolism (i.e., when gas blockage occurs in the xylem conduits reducing water transport from roots to leaves; Tyree and Zimmermann 2002). The ability of trees to resist xylem embolism is strongly linked to their survival and recovery during and after a drought event (Choat et al. 2012, Babst et al. 2019). Species more vulnerable to xylem embolism face an elevated risk of mortality and habitat loss compared with more resistant species (Tyree and Zimmermann 2002, Ohlemüller et al. 2006, Czúcz et al. 2011). Another important trait associated with drought tolerance is the leaf turgor loss point (Ψ_tlp_, negative value), defined as the water potential (Ψ) at which leaf cells lose turgor pressure and can no longer maintain photosynthetic function (Bartlett et al. 2012). Species with more negative Ψ_tlp_ are typically more drought-tolerant, as they can sustain physiological activity at lower Ψ (McGregor et al. 2021). Generally, anisohydric species delay stomata closure, sustaining gas exchange at the cost of increased desiccation risk, while isohydric species maintain stable leaf Ψ through early stomatal closure (Tardieu and Simonneau 1998, Larcher 2003, McDowell et al. 2008). Drought responses have been widely studied, revealing impacts on leaf Ψ, turgor loss, photosynthesis, stomatal conductance, hydraulic traits, biomass allocation and nutrient uptake, and numerous studies are also available for temperate European species (e.g., Gessler et al. 2004, Aranda et al. 2015, Pflug et al. 2015, Salehi et al. 2020, Kahmen et al. 2022, Schumann et al. 2024, Ishimwe and Deligöz 2024). Overall, younger tree stages are at higher risk of drought stress as stored water reserves are smaller and the root system explores a smaller soil volume.

In tree nurseries and for afforestation purposes (Jacobs and Landis 2009, Çiçek et al. 2022), fertilization is often applied to improve growth and productivity (Schönbeck et al. 2020, Geremew et al. 2021). Fertilization effects may result from changes in the availability of several nutrients. Nitrogen (N) is closely linked to photosynthetic capacity, leaf development and biomass production (e.g., Evans and Clarke 2019), whereas potassium (K) plays an important role in stomatal regulation, osmoregulation and plant water relations (Wang et al. 2013). Phosphorus (P) may also influence growth and drought responses through its role in energy metabolism and root development (Vance et al. 2003). Accordingly, fertilization has been associated with increased photosynthetic capacity, higher stomatal conductance (Zhu et al. 2020), and enhanced height and diameter growth (Möhl et al. 2019). In parallel, it can lead to anatomical changes, such as increased vessel diameter (Spannl et al. 2016), improving water transport capacity (Medeiros et al. 2016). However, such changes may reduce drought resistance by increasing water demand and altering biomass allocation away from stress-tolerant structures (e.g., Plavcová et al. 2013, Beikircher et al. 2019, Feng et al. 2023). Thus, fertilization may simultaneously promote growth but also increase vulnerability under drought, which is particularly relevant under ongoing climate change and increasing drought frequency. Moreover, many European forest ecosystems, including those in Central Europe, are exposed to elevated nitrogen inputs (e.g., Rennenberg and Dannenmann 2015), which may stimulate growth but also induce nutrient imbalances and thereby modify tree responses to drought. This further highlights the importance of understanding fertilization effects on tree performance under drought.

Despite extensive research on tree responses to drought (e.g., Choat et al. 2018, Brodribb et al. 2020, McDowell et al. 2022*a*, 2022*b*), the role of nutrient availability and nutrient balance in drought responses during drought and post-drought remains insufficiently explored. Available studies have focused on the effects of fertilization under on short-term droughts, typically lasting days to months (Oddo et al. 2011, Luis et al. 2010, Faustino et al. 2013), while long-term impacts across repeated drought and post-drought phases remain less well understood. Rennenberg and Dannenmann (2015) reported that the amount of N taken up by mature trees during one growing season represents only 10% of their total N stock, but (although stored N reserves support early spring growth) active root uptake is essential during the main vegetation period when droughts are most likely to occur (Millard and Proe 1992). Impaired uptake during this period may limit regrowth of drought-damaged tissues (e.g., roots, stems, foliage). Ultimately, the ability of trees to acquire nutrients after drought depends on both soil nutrient dynamics and the tree’s physiological capacity to resume uptake (Gessler et al. 2017). Meanwhile, a few studies reported that fertilization may help to mitigate the adverse consequences of drought via influencing post-drought water relations (e.g., Wang et al. 2022, Chambi-Legoas et al. 2023, Marušic et al. 2023). Understanding how nutrient supply modulates tree resilience during drought and post-drought is therefore crucial, particularly for afforestation efforts that rely on young planting stock under increasingly variable climate conditions.

In this study, we investigated the interactive effects of fertilization and drought on gas exchange, hydraulic traits and growth in saplings of three ecologically and economically important Central European tree species (*Pinus sylvestris, Fagus sylvatica* and *Quercus robur*). We examined the physiological responses of fertilized and control trees during and after drought, aiming to address the following hypotheses: (i) responses to fertilization and drought with regard to water and carbon relations as well as growth are species-specific; (ii) fertilization weakens the drought resistance of saplings; and (iii) fertilization improves post-drought physiological performance and growth. By using a controlled pot experiment, this study should provide insights into the potential role of fertilization in modulating drought and post-drought responses of young trees, while also highlighting the need for subsequent field validation.

## Materials and methods

### Plant material and experiment design

**Figure 1:**
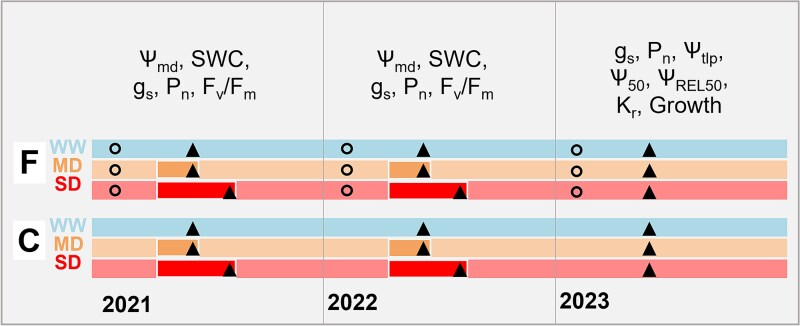
Experimental design. Saplings were exposed to two nutrient treatments, i.e., fertilized (F) and control (C) and three water regimes: well-watered (WW, blue), moderate drought (MD, orange) and severe drought (SD, red). Circles indicate annual fertilization, dark colored boxes represent drought treatments and solid triangles mark measurement times. Details are given in the text. Abbreviations: Ѱ_md_, midday water potential; SWC, water soil content; g_s_, stomatal conductance; P_n_, net photosynthesis; F_v_/F_m_, chlorophyll fluorescence; Ѱ_tlp_, turgor loss point; Ѱ_50_, embolism resistance of stem; Ѱ_REL50_, drought tolerance of leaf tissue; K_r_, root hydraulic conductivity.

Experiments were conducted on three European forest species: *P. sylvestris* L. (evergreen, coniferous), *F. sylvatica* L. (deciduous, diffuse-porous) and *Q. robur* L. (deciduous, ring-porous). Hereafter, these species are referred to as pine, beech and oak, respectively. In autumn 2020, a total of 360 saplings (120 per species), 30–50 cm in height, were purchased from the ‘Tiroler Landesforstgärten’ and planted in 2 L square tapered pots (10 × 13 × 15 cm, bottom width × top width × height) filled with a standard soil mixture designed for alpine plants (leaf mold:ground soil:coconut fiber:sand:horticultural lava:rock meal; 5:2:2:2:1:0.15). In spring 2022, due to root growth, all saplings were transplanted into 4 L round tapered pots (13 × 22 × 18.5 cm, bottom diameter × top diameter × height) using the same soil mixture. The pot size was selected according to standard nursery practice with respect to sapling size and age, and the experiment ended before another transfer to larger pots would have been necessary. Therefore, rooting space was not expected to substantially limit sapling development. The soil provided sufficient nutrients for growth of the saplings over the 3 years of the study, and no additional fertilization was necessary for normal growth and development. The pots were arranged in a randomized design at the Innsbruck Botanical Garden (affiliated with the Department of Botany, University of Innsbruck) and repositioned monthly to minimize edge effects. Regular watering was maintained to prevent dehydration. In summers, the pots were moved to a glasshouse for drought treatments. A brief diagram of the experimental design is shown in Figure 1.

In spring 2021, saplings of each species were divided into two groups and fertilized with 0 g (control, C) or 19 g (fertilized, F) standard nitrogen–phosphorus–potassium (NPK) fertilizer per pot. Because pine is a gymnosperm and differs in nutrient requirements from the two angiosperm species, commercially available fertilizers commonly applied for the respective plant types were used. This approach was chosen to simulate a simple annual fertilization treatment based on standard nursery practice. Pine received COMPO conifer fertilizer (COMPO GmbH, Germany) containing 16% N and 13.5% K_2_O, while beech and oak were fertilized with COMPO standard fertilizer containing 7% N, 3% P_2_O_5_ and 3% K_2_O (in 2021 and 2023), and GPI fertilizer (GPI green partners international GmbH&Co. KG, Germany) containing 9% N, 4% P_2_O_5_ and 7% K_2_O (in 2022; depending on availability of fertilizer).

In summer 2021, saplings from each fertilization group and species were further divided and subjected to three water regimes: (i) well-watered (WW, *n* = 20), saplings were watered regularly to avoid dehydration; (ii) moderate-drought (MD, *n* = 20), saplings were exposed to a controlled drought stress by stopping irrigation until plants reached a water potential (Ѱ) inducing 30% (conifer, ca 7–8 days) and 50% (angiosperms, ca 4–5 days) loss of hydraulic conductivity (LC, according to previous vulnerability analyses, Feng et al. 2026; Lobo et al. 2018); and (iii) severe-drought (SD, *n* = 20), saplings were dehydrated to Ѱ at which 70% (conifer, 21–22 days) and 80% (angiosperms, 9–10 days) LC were induced. Once the saplings had reached the target Ѱ, they were subjected to a series of measurements (*n* = 5–10 pots per water regime, fertilization and species), including soil water content (SWC), stomatal conductance (g_s_), net photosynthesis (P_n_) and chlorophyll fluorescence (F_v_/F_m_). After the measurements, the pots were re-irrigated and kept well-watered for further measurements in the following years.

In spring 2022, saplings were fertilized again (0 g C, 19 g F; see above), followed by a second drought-irrigation cycle in summer. As in 2021, 5–10 pots (per water regime, fertilization and species) were randomly selected for the same physiological measurements.

In spring 2023, fertilization was repeated (0 g C, 19 g F; see above), and in summer, the saplings (*n* = 5–10 per water regime, fertilization and species) were used for a series of measurements, including leaf tissue nutrient concentrations and soil N contents, gas exchange (g_s_, P_n_), hydraulics (pressure-volume curves, embolism resistance of stems, stem-specific conductivity, drought resistance of leaf tissues, root hydraulic conductance) and growth (sapling height, stem diameter, specific leaf area, biomass, root-to-shoot ratio).

### Midday water potential (Ѱ_md_), soil water content (SWC) and chlorophyll fluorescence (F_v_/F_m_)

To assess the water status, midday water potential (Ѱ_md_) was measured at ~12:30 h on end twigs (pine; ca 5–10 cm long) or leaves (beech, oak) (*n* = 5–10) using a Scholander pressure chamber (1505D, PMS Instrument Company, USA). Before excision, the samples were enclosed in aluminum zipper bags for 30 min to equilibrate. Ѱ_md_ was monitored every other day and, once it approached the target value, it was measured daily. Once the target Ѱ_md_ was reached, soil water content (SWC) was measured using a handheld soil moisture sensor (HydroSense II, Campbell Scientific Ltd, Australia), with the probe inserted vertically to its full rod length (12 cm) into the center of each pot (*n* = 5–10) to avoid contact with the pot wall.

Chlorophyll fluorescence (F_v_/F_m_) was also measured at the target Ѱ_md_ to assess photochemical efficiency. Leaves (*n* = 5–10) were covered with leaf clips and dark-adapted for 30 min before measurements were taken using a portable fluorometer (PEA MK2, Hansatech Instruments, UK).

### Leaf tissue nutrient concentrations and soil N content

Leaves of C and F saplings from WW treatment were collected for nutrient analysis in 2023. For each species and fertilization treatment (*n* = 3), leaves were oven-dried at 80° C for 48 h. Subsequently, 5 g of dried leaf material were collected and ground using a TissueLyser II (QIAGEN, Germany). The ground samples were sent to the Austrian Research Centre for Forests (Vienna, Austria) for determination of N, P and K concentrations. Nutrient concentrations are expressed as percentage of leaf dry mass. Soil N content data from the same experimental setup were taken from Feng et al. (2026) to provide additional information on soil nutrient availability.

### Stomatal conductance (g_s_) and net photosynthesis (P_n_)

Stomatal conductance (g_s_) and net photosynthesis (P_n_) measurements were conducted on sunny days between 12:00 and 15:00 h on saplings which had reached target Ѱ during drought periods in 2021 and 2022 (*n* = 5–10), as well as post-drought in 2023 (*n* = 3–8). Fully mature leaves were attached to a gas exchange system (Li-6400 in 2021 and 2022, and Li-6800 in 2023; both LI-COR Environmental GmbH, USA) with measurement parameters set to ambient temperature, humidity and CO_2_ concentration and an airflow rate of 500 μmol s^−1^. Preliminary tests were conducted to determine light saturation points, resulting in optimal photosynthetically active radiation (PAR) levels of 2000 μmol m^−2^ s^−1^ for pine and beech, and 1000 μmol m^−2^ s^−1^ for oak. Data were recorded once g_s_ and P_n_ stabilized (ca 3 min).

### Pressure-volume curves and turgor loss point (Ψ_tlp_) determination

Pressure-volume curves (pv curves) describe the relationship between the leaf water potential (inversely transformed, 1/Ψ) and relative water deficit (RWD) of plant tissues and enable determination of the water potential at turgor loss point (Ψ_tlp_). Pots were well watered the evening before the measurement to ensure full saturation. Twigs of pine (*n* = 5) or leaves of beech and oak (*n* = 5) were cut the next morning (ca 08:00 h) and immediately weighed to determine the turgid weight (TW) using an analytical balance (BP61S, Sartorius AG, Germany). The twigs or leaves were then dehydrated on a bench, and periodic fresh weight (FW) measurements were taken at different Ψ (measured as above). Once Ψ reached −4 to 5 MPa, samples were oven-dried at 80 °C for 48 h to determine the dry weight (DW), and the RWD was calculated as:


$$RWD=100-100\times \frac{FW- DW}{TW- DW}$$

The R package ‘pvldcurve’ (Raesch 2020) was used to construct pv curves and determine Ψ_tlp_ for each individual twig and leaf, respectively.

### Embolism resistance of stems (Ψ_50_) and specific stem conductivity (k_s_)

Embolism resistance of stems was compared via assessing the Ψ at 50% loss of conductivity (Ψ_50_), which was calculated from either vulnerability curves (VCs, beech, pine, *n* = 4–6) or extraction curves (ECs, oak, *n* = 3–5).

Vulnerability curves were constructed using the Cavitron method (Cochard 2002, Cochard et al. 2005, Beikircher et al. 2010). Briefly, a 27-cm-long stem segment was excised underwater, with side branches and leaves removed. The distal and basal ends (ca 5 cm) were debarked before placing the segment into a custom-built rotor of a modified centrifuge (Sorvall RC-5, Thermo Fisher Scientific, USA). Both ends of the segment remained submerged in reservoirs filled with distilled, filtered and degassed water containing 0.005% (v/v) Micropur Forte MF 1000F (Katadyn Products, Switzerland). The reservoirs had spill holes positioned 10 mm and 16 mm from the base, respectively. While spinning in the centrifuge, a pressure gradient was generated across the segment due to difference in water levels between the two reservoirs. This gradient allowed for the measurement of k_s_, which was calculated based on stem length and cross-sectional area using a standard regression method (Wang et al. 2014). The segment was first spun at an RPM corresponding to a Ψ of −0.25 MPa and equilibrated for 10–15 min before the initial k_s_ was measured. Subsequent k_s_ measurements were taken stepwise as RPM increased, inducing Ψ reductions of ca −0.5 MPa per step, until LC exceeded 95%. The R package fitplc (Duursma and Choat 2017) was used to fit VCs using the Weibull function and determine Ψ_50_ for each sample.

Extraction curves measurements were conducted using a ChinaTron centrifuge (H2100R, Cence, China) equipped with a digital camera (Scout Sc640gm, Basler AG, Germany) and a C-mount lens (HF16 HA-1B, Fujinon Corp., Japan). The camera system enabled image acquisition to track meniscus positions in the reservoirs. The associated software recorded RPM values, pixel positions of the extracted water meniscus over time, and saved all relevant data to a text file. Stem segment preparation, water handling and reservoir setup followed the same protocol as for VCs measurements, except that the reservoirs used for ECs had no spill holes. The segment was placed in the rotor with both ends submerged in reservoirs filled to a consistent water height of 1 cm. The segment was first spun at 800 RPM (≈−0.08 MPa) for 10–20 min to allow equilibration. Once menisci in both reservoirs stopped moving and merged into a single meniscus (confirmed by real-time imaging from the digital camera), the software was activated and the initial pixel position of the meniscus was recorded as zero. Subsequently, the rotational speed, expressed in revolutions per minute (RPM), was increased in steps of 0.5 MPa from −0.1 to −6.1 MPa. At each Ψ, initially, the meniscus moved radially toward the axis of rotation due to water extraction from the segment, causing a gradual increase in pixel position over 10–30 min, followed by a slight decline in pixel position due to minor water loss from evaporation. This process was repeated until the final Ψ of −6.1 MPa was reached. The R package fitplc (Duursma and Choat 2017) was used to fit ECs using the Weibull function, and Ψ_50_ was determined (Peng et al. 2019, Du et al. 2019). Unlike VC measurements, k_s_ determination was not possible in EC measurements.

### Drought resistance of leaf tissues (Ѱ_REL50_)

The electrolyte leakage method (Morin et al. 2007, Ganthaler and Mayr 2015) was used to assess drought resistance of leaf tissues by quantifying electrolyte leakage from the symplast to the apoplast due to cellular damage. This method involves measuring the electrical conductivity of a solution containing leaf samples, with the conductivity of boiled samples (to ensure that all cells have been killed) used as a reference for total tissue electrolyte content. Saplings were well watered the evening prior to measurement. Leaves were harvested in the morning and dehydrated on the bench to different Ψ between 0 and −7 MPa. At different Ψ, 15 needle segments (5 mm in length, pine) were excised per sample using a razor blade, or 10 circular leaf discs (5 mm in diameter, beech and oak) were obtained per sample using a cork borer, and placed in a 2 mL Eppendorf safe-lock tube containing 1.5 mL distilled water. Tubes were placed in a shaker with thermoregulation (Thermomixer C, Eppendorf, Germany) for 1 h at 25°C. The electrical conductivity (C_1_) was measured with a conductivity meter (LAQUAtwin EC-22, Horiba, Japan). Then the tubes were heated to 100°C for 30 min, followed by an additional 30 min of shaking at 25°C before the electrical conductivity (C_2_) was measured. The relative electrolyte leakage (REL) was calculated as:


$$REL=\frac{C_1-{C}_0}{C_2-{C}_0}\times 100$$

where C_0_ represents the conductivity of control samples (Ψ between 0 and −0.5 MPa, i.e., nearly fully hydrated leaves).

Data (REL vs Ψ) were pooled (up to 40 measurements per water regime, fertilization and species, respectively) to construct leakage curves. Curve fitting was performed using the Weibull function in the R package ‘fitplc’ (Duursma and Choat 2017), and Ψ_REL50_ (the Ψ at 50% REL) was determined. In all species, maximum leakage due to dehydration was lower than that observed after boiling, likely due to partial retention of cellular contents within the tissue. Consequently, the endpoint of the fitted curves was set to 100%, and all values were adjusted accordingly.

### Root hydraulic conductance (K_r_)

Root hydraulic conductance (K_r_) was measured using a high-pressure flow meter (HPFM Gen 3; Dynamax, Houston, TX, USA), following the protocol described by Losso et al. (2016). The base of the stem was tightly sealed in a plastic bag to create a water-filled receptacle, to allow the stem to be cut underwater, ca 5–10 cm above the soil surface. While still underwater, the cut surface was then trimmed several times with a razor blade to make it flat and clean, and the surrounding bark was carefully removed. Subsequently, the prepared stem was connected to the HPFM and perfused with measuring solution (same as for VCs measurements). Immediately afterward, three to five transient measurements were done (see Tyree et al. 1995), and the hydraulic conductance of the root system was calculated and then normalized to the stem xylem cross-sectional area (kg s^−1^ MPa^−1^ m^−2^; see Losso et al. 2016).

### Sapling height (H), stem diameter (D), specific leaf area (SLA), biomass and root to shoot ratio (root:shoot)

Sapling height (H, *n* = 8–10) was measured using a measuring tape, while stem diameter (D, *n* = 8–10) was recorded 1 cm above the soil surface using a caliper. For specific leaf area (SLA) determination, two needle bundles (pine) or two leaves (beech, oak) were collected per sapling (*n* = 5–8). Leaves were photographed alongside a scale, and leaf area was analyzed using ImageJ (Fiji, v2.15.1). The samples were then oven-dried at 80° C for 48 h. The SLA was calculated as dry weight divided by corresponding leaf area.

Saplings (*n* = 3–6) were separated into roots, stems and leaves. Each component was oven-dried at 80° C for 48 h and then weighed using a precision balance (0.01 g accuracy; ED4202S, Sartorius AG, Germany) to obtain the biomass. Root:shoot was calculated based on the dry weight of roots relative to the combined weight of stems and leaves.

### Statistical analysis

All analyses were conducted in R version 4.2.1. Differences between water regime of each fertilization group and species were tested with one-way ANOVA followed by pairwise comparisons using Tukey's honestly significant difference (HSD) test, after testing data for normality (Shapiro–Wilk test) and homoscedasticity (Levene test). All tests were performed at a probability level of 5% and data is presented as mean ± SE. For each species, a principal component analysis (PCA) based on the traits measured post-drought was conducted to examine patterns of trait covariation and to evaluate potential clustering of saplings according to fertilization and water regimes.

## Results

### Hydraulic and gas exchange parameters during drought

In WW saplings, Ѱ_md_ of all three species ranged between −0.5 and −1.1 MPa (Table 1). In MD treatment, pine and beech were dehydrated to −2.5 ± 0.1 and −2.4 ± 0.1 MPa, while oak reached −3.3 ± 0.1 MPa in C and −3.6 ± 0.1 MPa in F. In SD treatment, pine was dehydrated to a minimum of –3.6 ± 0.1 MPa, beech to −4.5 ± 0.1 MPa and oak to −5.1 ± 0.1 MPa. Accordingly, SWC varied between ca 20% (WW) and 0.1% (SD).

**Table 1:** Midday water potential (Ѱ_md_) and water soil content (SWC) of control (C) and fertilized (F) saplings of *P. sylvestris, F. sylvatica* and *Q. robur* under three water regimes: well-watered (WW), moderate drought (MD) and severe drought (SD) during the summer drought phases. Values represent mean ± SE. Different letters indicate significant differences among water regimes within the same fertilization group (*P* < 0.05).

Index	Treatment	*P. sylvestris*			*F. sylvatica*			*Q. robur*		
		WW	MD	SD	WW	MD	SD	WW	MD	SD
Ѱ_md_, MPa	C	−0.5 ± 0.2^a^	−2.5 ± 0.1^b^	−3.6 ± 0.1^c^	−0.9 ± 0.3^a^	−2.4 ± 0.1^b^	−4.5 ± 0.1^c^	−1.1 ± 0.1^a^	−3.3 ± 0.1^b^	−5.0 ± 0.1^c^
(*n* = 5–10)	F	−0.6 ± 0.1^a^	−2.5 ± 0.1^b^	−3.5 ± 0.1^c^	−1.1 ± 0.1^a^	−2.4 ± 0.1^b^	−4.4 ± 0.1^c^	−1.1 ± 0.1^a^	−3.6 ± 0.1^b^	−5.1 ± 0.1^c^
SWC, %	C	20.4 ± 1.1^a^	1.1 ± 0.1^b^	0.2 ± 0.1^c^	18.4 ± 1.4^a^	7.9 ± 0.2^b^	2.5 ± 1.2^c^	20.2 ± 0.1^a^	1.9 ± 0.3^b^	1.3 ± 0.4^b^
(n = 5–10)	F	19.9 ± 1.0^a^	0.8 ± 0.1^b^	0.1 ± 0.1^c^	19.1 ± 0.7^a^	7.6 ± 1.5^b^	1.5 ± 0.1^c^	18.5 ± 0.8^a^	1.4 ± 0.2^b^	0.9 ± 0.2^b^

**Figure 2:**
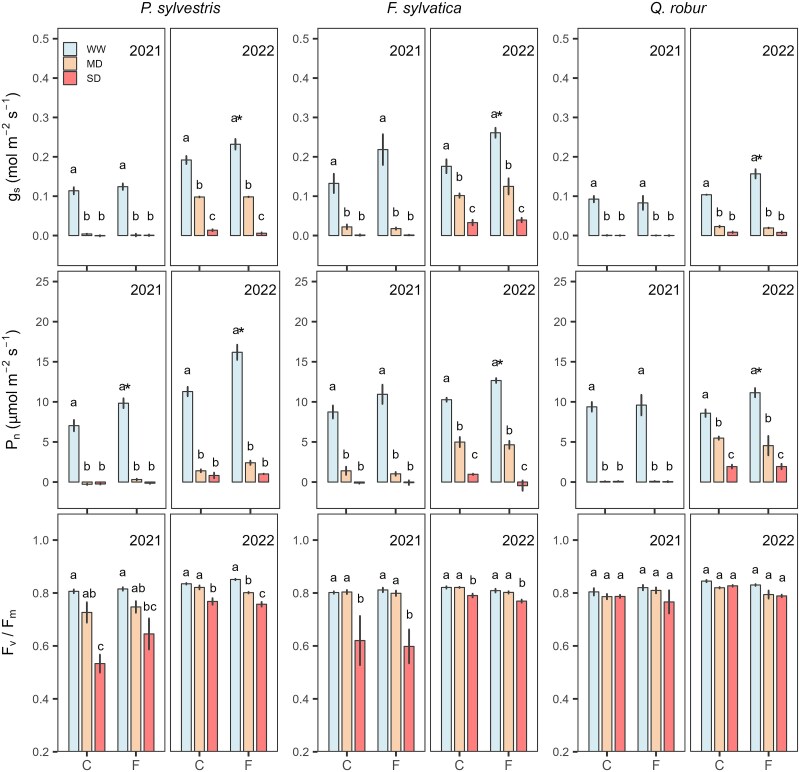
Stomatal conductance (g_s_), net photosynthesis (P_n_) and chlorophyll fluorescence (F_v_/F_m_) measured in control (C) and fertilized (F) *P. sylvestris, F. sylvatica* and *Q. robur* saplings under well-watered (WW, blue), moderate drought (MD, orange) and severe drought (SD, red) in summer of 2021 and 2022. Values represent mean ± SE, *n* = 5–10. Different letters indicate significant differences among saplings of WW, MD and SD within the same fertilization group (*P* < 0.05), while ‘^*^’ indicates significant differences between C and F saplings within the same water regimes (*P* < 0.05).

Under WW, F saplings of all species exhibited 20.8–44.4% higher g_s_ and P_n_ from 2021 to 2022 compared with C saplings (Figure 2). In 2021, g_s_ and P_n_ under both MD and SD rapidly declined to nearly zero in all three species, regardless of fertilization. A reduction in F_v_/F_m_ was also observed in both C and F saplings of pine and beech exposed to SD (from 0.81 ± 0.01 to 0.53 ± 0.03 and from 0.80 ± 0.01 to 0.62 ± 0.09, respectively), whereas oak remained unaffected (~0.8). In 2022, g_s_ and P_n_ under MD were higher than in 2021 for all three species, regardless of fertilization, while g_s_ and P_n_ under SD remained suppressed. Again, a reduction (though small) in F_v_/F_m_ was observed in pine and beech under SD (from 0.84 ± 0.01 to 0.70 ± 0.02 and from 0.82 ± 0.02 to 0.74 ± 0.02, respectively), regardless of fertilization, while it remained unchanged in oak (~0.8).

### Post-drought responses

#### Leaf tissue nutrient concentrations and soil N content

The F saplings showed higher tissue N concentrations than C saplings in all three species (Table S1 available as Supplementary Data at *Tree Physiology* Online). In pine, N increased from 1.31 ± 0.03% in C to 1.79 ± 0.13% in F saplings. In beech, N increased from 1.40 ± 0.25% to 2.29 ± 0.13%, and in oak from 1.42 ± 0.14% to 1.94 ± 0.02%. Tissue P concentrations were slightly higher in F compared with C saplings of beech and oak, increasing from 0.22 ± 0.01% to 0.29 ± 0.05% and from 0.24 ± 0.03% to 0.28 ± 0.02%, respectively, while only a minor increase was observed in pine. Tissue K concentrations were higher in F saplings of pine, from 0.73 ± 0.04% to 0.94 ± 0.05%, whereas K remained unchanged in beech and oak. Soil N content was also higher in F than C pots in all three species, increasing from 0.23 ± 0.01% to 0.30 ± 0.02% in pine, from 0.26 ± 0.01% to 0.35 ± 0.02% in beech, and from 0.24 ± 0.01% to 0.36 ± 0.01% in oak.

#### Gas exchange

**Figure 3:**
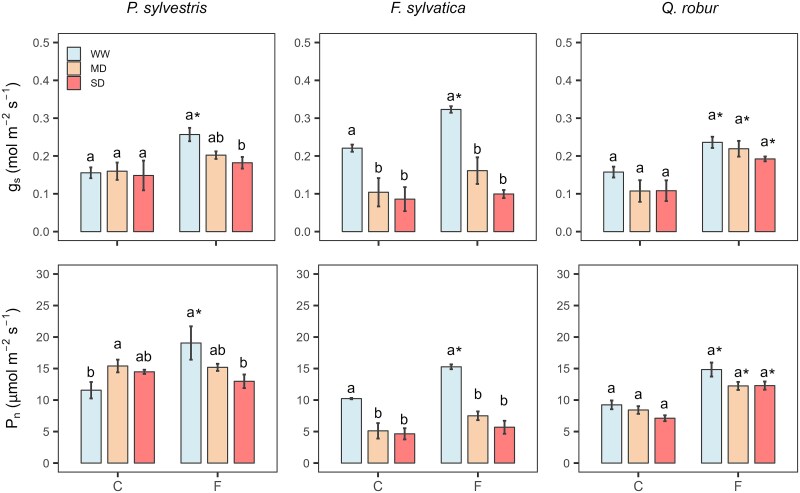
Stomatal conductance (g_s_) and net photosynthesis (P_n_) measured on control (C) and fertilized (F) *P. sylvestris, F. sylvatica* and *Q. robur* saplings in 2023 after two previous drought cycles (summer 2021 and 2022) with three different water regimes (i.e., blue: well-watered, WW; orange: moderate-drought, MD; red: severe-drought, SD). Values represent mean ± SE, *n* = 3–8. Different letters indicate significant differences among saplings of WW, MD and SD within the same fertilization group (*P* < 0.05), while ‘^*^’ indicates significant differences between C and F saplings within the same water regimes (*P* < 0.05).

In all WW species, F saplings exhibited increases in g_s_ and P_n_ by 45–65% compared with C saplings (Figure 3). In pine, C saplings maintained stable g_s_ levels, whereas F saplings showed a decline from 0.26 ± 0.02 (WW) to 0.18 ± 0.01 mol m^−2^ s^−1^ (SD). Similarly, P_n_ of F saplings decreased from 19.1 ± 2.64 (WW) to 13.0 ± 1.08 μmol m^−2^ s^−1^ (SD). In beech, regardless of fertilization, saplings exhibited a decrease in both g_s_ (to 0.10 ± 0.01 mol m^−2^ s^−1^) and P_n_ (to 5.68 ± 1.03 μmol m^−2^ s^−1^) under MD and SD compared with WW. For oak, both C and F saplings maintained relatively stable g_s_ and P_n_ across treatments with overall higher values in F. Mean g_s_ were 0.15 ± 0.01 mol m^−2^ s^−1^ in C and 0.23 ± 0.05 mol m^−2^ s^−1^ in F. Corresponding P_n_ were 9.24 ± 0.69 μmol m^−2^ s^−1^in C and 14.8 ± 1.11 μmol m^−2^ s^−1^ in F.

#### Hydraulics

##### Turgor loss point

**Figure 4:**
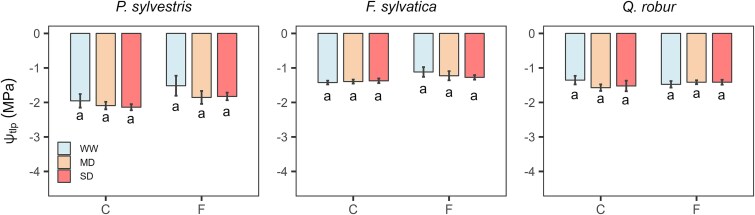
Turgor loss point (Ѱ_tlp_) of twigs (*P. sylvestris*) and leaves (*F. sylvatica, Q. robur*) of control (C) and fertilized (F) saplings measured in 2023 after two previous drought cycles (summer 2021 and 2022) with three different water regimes (i.e., blue: well-watered, WW; orange: moderate-drought, MD; red: severe-drought, SD). Values represent mean ± SE, *n* = 5. Different letters indicate significant differences among saplings of WW, MD and SD within the same fertilization group (*P* < 0.05), no difference was found between C and F saplings within a given water regime.

Ψ_tlp_ remained unchanged across fertilization and water regimes in all three species (Figure 4). Ψ_tlp_ ranged from −2.0 to −1.5 MPa in pine and from −1.4 to −0.9 MPa in beech and oak, showing minimal variation across fertilization groups and water regimes.

##### Embolism resistance and hydraulic conductivity

**Figure 5:**
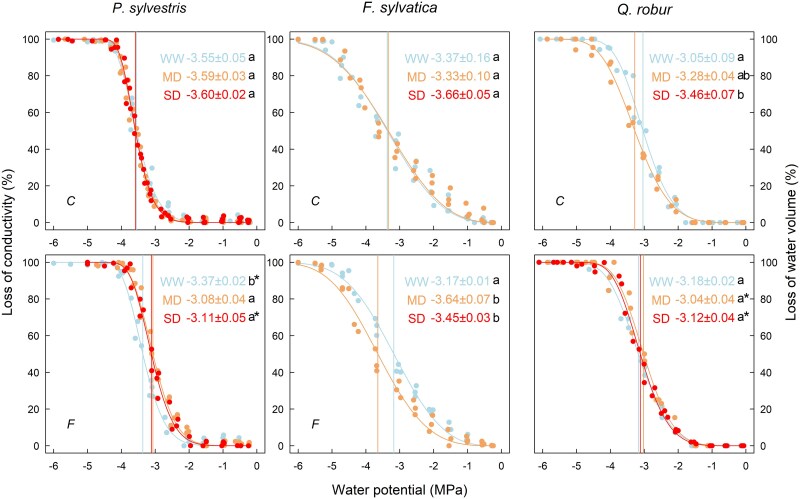
Vulnerability curves of *P. sylvestris* and *F. sylvatica*, and extraction curves of *Q. robur* measured on control (C, upper panels) and fertilized (F, lower panels) saplings in 2023 after two previous drought cycles (summer 2021 and 2022) with three different water regimes (i.e., blue: well-watered, WW; orange: moderate-drought, MD; red: severe-drought, SD). Embolism resistance, indicated by Ѱ_50_ (xylem pressure at 50% loss of conductivity or water volume), is indicated by vertical lines, and Ѱ_50_ values (mean ± SE) are also given in the panels. Different letters indicate significant differences among saplings of WW, MD and SD within the same fertilization group, while ‘^*^’ indicates significant differences between C and F saplings within the same water regimes (*P* < 0.05, *n* = 4–6 for VCs, *n* = 3–5 for ECs).

In pine, F saplings showed overall less negative Ψ_50_ than C saplings (around −3.6 MPa), with this difference being significant in WW (−3.37 ± 0.02 MPa) and SD (−3.11 ± 0.05 MPa) (Figure 5). In beech, Ψ_50_ of C saplings overall remained around −3.5 MPa regardless of water regimes. But in F saplings, a decrease in Ψ_50_ was observed in both MD (−3.64 ± 0.07 MPa) and SD (−3.45 ± 0.03 MPa), compared with WW (−3.17 ± 0.01 MPa). In oak, Ψ_50_ of C saplings ranged from −3.05 ± 0.09 MPa (WW) to −3.46 ± 0.07 MPa (SD). In F saplings, Ψ_50_ remained close to −3.1 MPa and was less negative than that of C saplings under both MD (−3.04 ± 0.04 MPa) and SD (−3.12 ± 0.04 MPa) conditions.

Under WW, k_s_ showed no difference between C and F saplings of pine (Figure S1 available as Supplementary Data at *Tree Physiology* Online). k_s_ of C saplings remained unchanged across water regimes (averaging 5.5 × 10^−4^ kg MPa^−1^ s^−1^ m^−1^), while that of F saplings decreased from 6.04 ± 0.23 × 10^−4^ (WW) to 3.24 ± 0.90 × 10^−4^ kg MPa^−1^ s^−1^ m^−1^ (SD). In beech, F saplings (6.19 ± 0.20 × 10^−4^ kg MPa^−1^ s^−1^ m^−1^) under WW exhibited higher k_s_ than C saplings (3.69 ± 0.53 × 10^−4^ kg MPa^−1^ s^−1^ m^−1^). Among C saplings, k_s_ remained unaffected across water regimes, though data showed high variation. In contrast, k_s_ of F saplings showed a progressive decline with increasing drought severity, i.e., from 6.19 ± 0.20 × 10^−4^ (WW) to 0.72 ± 0.27 × 10^−4^ kg MPa^−1^ s^−1^ m^−1^(SD).

##### Drought resistance of living tissues

**Figure 6:**
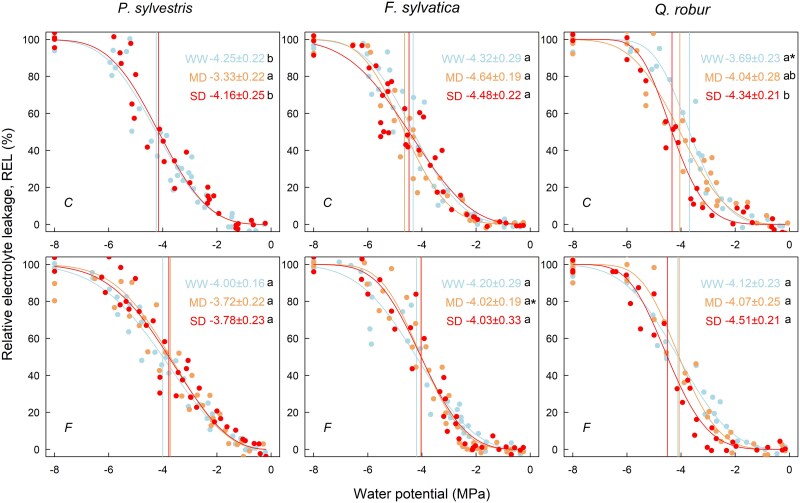
Relative electrolyte leakage (REL) versus water potential of leaves from control (C) and fertilized (F) saplings of *P. sylvestris, F. sylvatica* and *Q. robur* in 2023 after two previous drought cycles (summer 2021 and 2022) with three different water regimes (i.e., blue: well-watered, WW; orange: moderate-drought, MD; red: severe-drought, SD). Vertical lines indicate the water potential at 50% REL (Ѱ_REL50_), and values (mean ± SE) are also given in the panels. Different letters indicate significant differences among saplings of WW, MD and SD within the same fertilization group, while ‘^*^’ indicates significant differences between of C and F saplings within the same water regimes (*P* < 0.05, up to 40 samples per water regime, fertilization and species).

In WW pine and beech, Ψ_REL50_ did not differ between C and F saplings, while in oak Ψ_REL50_ showed a decrease of 0.43 MPa in F saplings compared with C saplings (Figure 6). In pine, REL data of C saplings showed high variability (from −4.25 ± 0.22 MPa in WW to −3.33 ± 0.22 MPa in SD). In F saplings, Ψ_REL50_ averaged at −3.8 MPa across all water regimes. Beech Ψ_REL50_ remained consistent across all water regimes, averaging −4.5 MPa in C and −4.0 MPa in F. The F saplings in MD exhibited a less negative Ψ_REL50_ (−4.02 ± 0.19 MPa), compared with C saplings (−4.64 ± 0.19 MPa). In oak, Ψ_REL50_ showed a decreasing trend across water regimes, with SD saplings in C exhibiting the most negative value at −4.34 ± 0.21 MPa.

##### Root hydraulic conductance

**Figure 7:**
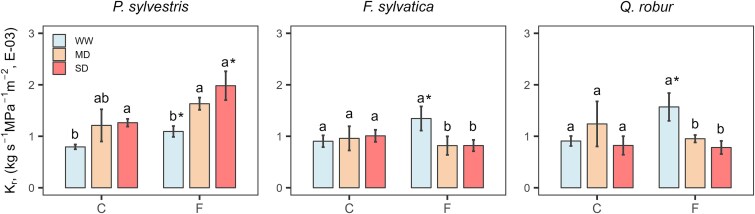
Root hydraulic conductance (K_r_) measured on control (C) and fertilized (F) saplings of *P. sylvestria, F. sylvatica* and *Q. robur* in 2023 after two previous drought cycles (summer 2021 and 2022) with three different water regimes (i.e., blue: well-watered, WW; orange: moderate-drought, MD; red: severe-drought, SD). Mean ± SE, *n* = 3–6. Different letters indicate significant differences among saplings of WW, MD and SD within the same fertilization group (*P* < 0.05), while ‘^*^’ indicates significant differences between C and F saplings within the same water regime (*P* < 0.05).

In all WW species, F saplings exhibited higher K_r_ than C saplings (Figure 7). An increasing trend in pine K_r_ was observed across water regimes in both C and F, with the highest K_r_ recorded in F saplings under SD (1.98 ± 0.28 × 10^−3^ kg s^−1^ MPa^−1^ m^−2^). For beech and oak, K_r_ remained unchanged across water regimes in C (both ca 1.0 × 10^−3^ kg s^−1^ MPa^−1^ m^−2^), while it decreased in both species under MD and SD (both ca 0.8 × 10^−3^ kg s^−1^ MPa^−1^ m^−2^) in F.

#### Growth

In WW saplings of C and F, no difference in H within pine and beech was observed, whereas oak exhibited taller saplings in F (105.0 ± 3.9 cm) than C (78.1 ± 3.0 cm) (Table S2 available as Supplementary Data at *Tree Physiology* Online). In C of all species, H remained consistent across water regimes, averaging ca 88.2 cm in pine, 62.9 cm in beech, and 82.7 cm in oak. In F saplings of pine and beech, H was reduced in SD (73.0 ± 9.4 cm for pine and 51.0 ± 2.5 cm for beech) compared with WW (97.0 ± 2.6 cm for pine and 69.5 ± 1.4 cm for beech). In oak, F saplings had similar H across water regimes.

In WW of oak, F saplings exhibited higher D (1.13 ± 0.07 cm) than C saplings (0.98 ± 0.05 cm) only, while no differences were observed in pine or beech. In pine, a reduction in D was observed in C saplings under SD (1.24 ± 0.06 cm) compared with WW (1.48 ± 0.05 cm), whereas F saplings remained unaffected. In beech, D in F saplings showed a decline under SD (1.07 ± 0.01 cm) compared with WW (1.24 ± 0.02 cm), while no change was observed in C saplings. In oak, D remained unchanged across water regimes in both C and F.

In WW of pine and beech, F saplings exhibited higher SLA than C saplings, while no difference was observed in oak (Table S2 available as Supplementary Data at *Tree Physiology* Online). In pine, SLA increased from 43.5 ± 3.1 in WW to 59.8 ± 1.8 cm^2^g^−1^ in SD in C. In contrast, in F saplings we observed a decline in SLA from 63.7 ± 4.4 in WW to 41.3 ± 3.2 cm^2^g^−1^ in SD, resulting in lower SLA in F than in C under SD. A similar pattern was observed in beech, where SLA in C increased from 190 ± 10.4 in WW to 247 ± 10.0 cm^2^g^−1^ in MD, whereas SLA in F decreased from 284 ± 6.4 (WW) to 245 ± 11.7 cm^2^g^−1^ (SD). In oak, SLA remained unchanged across water regimes in both C and F. However, F under SD (168 ± 4.1 cm^2^g^−1^) exhibited higher SLA than C (140 ± 2.5 cm^2^g^−1^).

**Figure 8:**
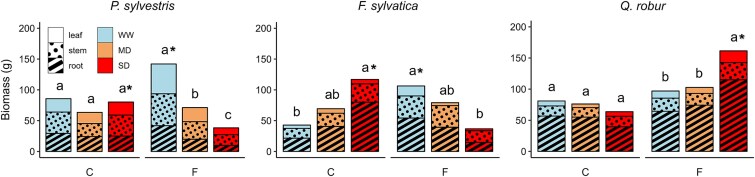
Biomass distribution among leaves, stems and roots in control (C) and fertilized (F) saplings of *P. sylvestris, F. sylvatica* and *Q. robur* in 2023 after two previous drought cycles (summer 2021 and 2022) with three different water regimes (i.e., blue: well-watered, WW; orange: moderate-drought, MD; red: severe-drought, SD). Different letters indicate significant differences among saplings of WW, MD and SD within the same fertilization group (*P* < 0.05, *n* = 3–6), while ‘^*^’ indicates significant differences between C and F saplings within the same water regime (*P* < 0.05, *n* = 3–6).

Under WW, F saplings of pine and beech exhibited greater total biomass compared with C saplings, while oak showed only a slight increase (Figure 8). In pine, total biomass in C remained stable across water regimes (averaging 80 g), whereas in F, it experienced a substantial reduction from 141 ± 24 g (WW) to 38 ± 7 g (SD). In beech, total biomass in C saplings increased from 43 ± 12 g (WW) to 117 ± 17 g (SD), while the opposite trend was observed in F saplings (106 ± 31 g in WW versus 37 ± 19 g in SD). In oak, total biomass in C saplings remained stable across water regimes (averaging 75 g), whereas F saplings showed a pronounced increase from 97 ± 2 g (WW) to 161 ± 16 g (SD), resulting in higher total biomass in F than C under SD.

#### PCA analysis

**Figure 9:**
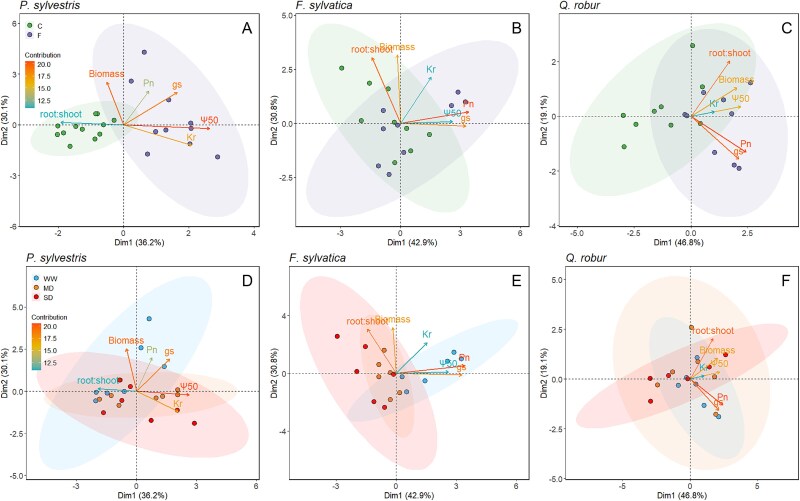
Principal component analysis (PCA) illustrating associations among measured traits (total biomass, Biomass; root:shoot; net photosynthesis, Pn; stomatal conductance, gs; stem embolism resistance, Ψ_50_; and root hydraulic conductance, K_r_) in the three studied species in 2023 post-drought. Shaded areas represent clusters of saplings grouped by fertilization treatment (A–C) or water regime (D–F). Arrow colors indicate each variable’s contribution (%) to the principal components, as shown in the color scale.

The relationships among measured parameters within each species were examined via PCA (Figure 9). In pine, the first two principal components explained 66.3% of the variance (Figure 9A). PCA revealed positive correlations between P_n_ and g_s_ (*P* < 0.05), g_s_ and Ψ_50_ (*P* < 0.05), and Ψ_50_ and K_r_ (*P* < 0.01), while total biomass was negatively correlated with K_r_ (*P* < 0.05) and root:shoot with Ψ_50_ (*P* < 0.01). The PCA clustering shows a distinct separation between F saplings (purple cluster, Figure 9A) and C saplings (green cluster, Figure 9A), which was predominantly associated with higher Ψ_50_ and lower root:shoot. No clear clustering by water regimes was observed (Figure 9D). In beech, the first two components explained 73.7% of the variance. Total biomass was positively correlated with root:shoot (*P* < 0.01), P_n_ with g_s_ (*P* < 0.001) and P_n_ with Ψ_50_ (*P* < 0.05). No clear separation was observed between C and F saplings (Figure 9B), whereas a clear cluster separation was observed between WW saplings (blue, Figure 9E) were separated from MD (orange) and SD (red, Figure 9E). In oak, the first two components explained 62.9% of the variance. Total biomass correlated positively with root:shoot (*P* < 0.05) and P_n_ (*P* < 0.05), root:shoot with Ψ_50_ (*P* < 0.05) and P_n_ with g_s_ (*P* < 0.001), and Ψ_50_ (*P* < 0.05). The clusters separation between fertilization treatments (Figure 9C) was not as strong as in pine but a partial difference between C and F can still be observed. No clear clustering was observed between water regimes (Figure 9F).

## Discussion

Fertilization generally promoted gas exchange, hydraulic conductance and biomass accumulation but responses were species-specific and did not always persist under drought. While fertilized oak maintained or even increased its biomass and physiological performance under drought, the benefits of fertilization for pine and beech diminished under increasing drought intensity. No consistent changes in drought resistance traits (e.g., turgor loss point, embolism resistance) were observed across species in response to fertilization, which may indicate limited hydraulic acclimation potential of saplings. In the following, observed effects in the first 2 years (under drought) and in the third year (post-drought) are discussed in detail.

### Responses during drought (years 2021 and 2022)

Fertilization enhanced g_s_ and P_n_ under well-watered (WW) conditions in all studied species (Figure 2), which is consistent with previous findings (e.g., Evans and Clarke 2019, Zhu et al. 2020). However, saplings exhibited species-specific responses to drought stress: in 2021, beech maintained low but measurable g_s_ and P_n_ under mild drought (MD), while both pine and oak had already closed their stomata and ceased photosynthesis. Under severe drought (SD), all three species exhibited complete stomata closure and no photosynthetic activity (Figure 2). These findings agree with the water management strategies of the species under study. Beech is often described as moderately anisohydric, modulating its g_s_ to sustain carbon assimilation under mild water stress (Tardieu and Simonneau 1998, Peuke et al. 2002). In contrast, both pine and oak are considered predominantly isohydric (Salmon et al. 2015, Thomsen et al. 2013), exhibiting more conservative water-use strategies and more sensitive stomatal control under declining soil water availability, which explains their early stomatal closure.

In 2022, the effects of drought were smaller for all three species with higher g_s_ and P_n_ than 2021, indicating acclimation to drought (best visible in MD plants, Figure 2). Such acclimation could be beneficial in terms of carbon assimilation, though maintaining a higher g_s_ under low Ѱ may increase the risk of hydraulic failure. Notably, in pine and beech, despite higher g_s_ (compared with 2021) P_n_ remained relatively small, which may indicate impaired PSII activity and possible photoinhibition or chlorophyll degradation (Gallé and Feller 2007) as indicated by the reduction in F_v_/F_m_ (Figure 2). In oak, F_v_/F_m_ remained overall stable, reflecting photochemical integrity during drought.

Overall, the drought responses of C and F saplings within each species were similar (Figure 2), suggesting that drought might be the main factor influencing gas exchange and photochemical efficiency. The negligible effect of fertilization on drought responses contrasts with some previous findings (e.g., Wang et al. 2022, Chambi-Legoas et al. 2023, Marušic et al. 2023) and our hypothesis that fertilized plants would perform better under drought (e.g., Kreuzwieser and Gessler 2010). The lack of fertilization effects under drought indicates limited physiological plasticity in saplings, which did not allow to profit from additional fertilization.

### Responses post-drought (year 2023)

After 3 years of fertilization, and 1 year after the second drought, fertilized WW plants showed higher g_s_ and P_n_ than control plants in all study species. Leaf nutrient analyses of WW saplings confirmed that fertilization altered plant nutritional status, particularly by increasing N concentrations in all three species. Fertilized saplings also showed higher K concentrations in pine, whereas P concentrations were not significant among species. Higher soil N contents in fertilized pots further indicated that the fertilization treatment increased nutrient availability in the substrate. Transplanting into larger pots in 2022 may have increased baseline nutrient availability for all saplings. However, because all treatments were transplanted using the same soil mixture, this effect should have applied equally to control and fertilized plants. Moreover, leaf nutrient analyses at the final harvest showed higher N concentrations in fertilized saplings of all species, indicating that treatment differences in nutritional status persisted despite the additional substrate. Therefore, the observed fertilization effects on gas exchange, hydraulic conductance and growth can be linked to an actual improvement in leaf nutrient status. This agrees with findings from previous studies (e.g., Evans and Clarke 2019, Zhu et al. 2020), as the improved nutrient status improved leaf development, chlorophyll content and photosynthetic enzyme activity (Nasar et al. 2022). However, increased nutrient availability may not always be beneficial, because elevated nitrogen supply, particularly when nutrient availability is not fully balanced, could also contribute to nutritional imbalances and partly explain why fertilization benefits were not consistently maintained under drought. An important question was therefore whether saplings that had experienced drought in two previous summer seasons still benefited from fertilization during the post-drought phase. Once again, the response was species-specific: following the drought, both pine and beech F saplings did not show any differences in P_n_ and g_s_ when compared with C saplings (MD and SD in Figure 3), indicating that positive fertilization effects were compensated by negative drought effects. Accordingly, a decreasing SLA with increasing drought stress was found in F saplings (Table S2 available as Supplementary Data at *Tree Physiology* Online). This differs with some studies that reported improved recovery in fertilized beech (Gessler et al. 2017, Marušic et al. 2023) and might depend on the extent of fertilization and drought stress, and the developmental stage of plants. In our experiment, only oak profited from fertilization after repeated droughts. Though lower than in WW saplings, P_n_ and g_s_ of both MD and SD trees were up to 40% higher in F compared with C saplings. This observation supports previous reports of oak’s drought tolerance (Niemczyk et al. 2024).

We found no consistent shifts in osmoregulation (Ψ_tlp_, Figure 4), cellular resistance to dehydration (Ψ_REL50_, Figure 6) or embolism resistance (Ψ_50_, Figure 5), neither upon fertilization nor after drought treatments. This was unexpected, given that nutrient availability, particularly K, are known to play a vital role in osmoregulation, and their availability can influence the ability of plants to maintain turgor, potentially altering Ψ_tlp_ (Wang et al. 2013). We acknowledge that the Ψ_tlp_ values measured here, particularly in beech and oak, were higher (i.e., less negative) than several published values for these species. For instance, a meta-analysis of Ψ_tlp_ conducted in Central European forests (Kunert and Tomaskova 2020) has reported mean Ψ_tlp_ values of −2.5 to −2.0 MPa for beech and oak (−1.4 to −0.9 MPa in the present study, Figure 4), and approximately −2 MPa for pine (−2 to −1.5 MPa in the present study, Figure 4). These comparatively high Ψ_tlp_ values may limit the interpretation of subtle fertilization effects on drought vulnerability. Therefore, the absence of clear treatment effects on Ψ_tlp_ should be interpreted with caution, particularly because measurements were conducted on young saplings, and these measurements were conducted during the 2023 recovery phase rather than at peak drought. We only found a small decrease in Ψ_50_, i.e., increase hydraulic safety, in oak (see C saplings in Figure 6), but no drought-induced shifts in the other species, and fertilization-related shifts were completely absent. This is in contradiction to some previous studies, which found increased resistance (e.g., Faustino et al. 2013, Goldstein et al. 2013) after fertilization treatments, while others have reported decreased resistance (e.g., Zhang et al. 2018, Beikircher et al. 2019). Also, drought was suggested to cause the formation of narrower xylem conduits, thereby enhancing embolism resistance and reducing the risk of hydraulic failure (Beikircher and Mayr 2009, Lens et al. 2022). These discrepancies might be explained by a limited plasticity or a lack of acclimation potential in earlier ontogenetic stages in Ψ_tlp_, Ψ_REL50_ and Ψ_50_, as well as by differences in drought duration or intensity.

In contrast to the aboveground hydraulic traits, root hydraulics showed pronounced, yet species-specific, changes in response to fertilization and drought: all species exhibited an increased root conductivity (K_r_) following fertilization (see WW plants in Figure 7). This enhancement may be attributed to increased conduit dimensions and/or fine root growth, which reduce resistance to water flow and support higher transpiration (e.g., Trubat et al. 2006, 2012, Beikircher et al. 2019, Tyree and Zimmermann 2002). In pine, we also found an increased K_r_ after drought in C and even more in F saplings (Figure 7), which might have been related to compensatory root growth, changes in root xylem anatomy, and/or enhanced aquaporin activity aimed at restoring water uptake capacity (e.g., Liu et al. 2014). In contrast, beech and oak did not benefit from fertilization when exposed to drought.

Fertilization caused higher total biomass production, stem height and diameter (see WW saplings in Figure 8, Table S2 available as Supplementary Data at *Tree Physiology* Online) in all species. This is in line with the known growth-promoting effects of fertilization through enhanced nutrient uptake, photosynthetic capacity and hydraulic transport (Figures 3 and 7, and Figure S1 available as Supplementary Data at *Tree Physiology* Online, e.g., Beikircher et al. 2019). In contrast, fertilized saplings showed pronounced growth reductions when exposed to additional drought stress: In pine and beech, growth limitation by drought stress dominated over positive fertilization effects, leading to reduced biomass, especially under SD treatment (Figure 8). Interestingly, the biomass of C pine saplings was not affected by drought. Accordingly, g_s_, P_n_, K_r_, k_s_ and root:shoot were also unimpacted (Figures 3 and 7, and Figure S1 available as Supplementary Data at *Tree Physiology* Online; Table S2 available as Supplementary Data at *Tree Physiology* Online), suggesting a conservative growth strategy. Conversely, C saplings of beech even exhibited an increase of biomass under drought, due to stimulated root growth (e.g., Schall et al. 2012, Zang et al. 2014). This was obviously not present in F saplings, probably because the high nutrient availability overruled growth signals.

Oak displayed the most robust growth post-drought. While C saplings maintained relatively stable in total biomass, F saplings increased biomass even under SD. This suggests that oak more effectively used added nutrients, which agrees with the observed high gas exchange (Figure 3) and investment into roots (Table S2 available as Supplementary Data at *Tree Physiology* Online). This also demonstrates that both, responses during and after drought have to be considered when characterizing species-specific fertilization effects. While responses of oak were similar to pine and beech for most traits analyzed during drought, differences were pronounced post-drought.

## Conclusion

The effects of fertilization and/or drought were species-specific, as revealed by the PCA analysis (Figure 9). The fertilization responses were overall stronger than drought effects in pine, and less pronounced in oak. Conversely, beech showed the strongest drought responses. Leaf nutrient analyses confirmed that fertilization altered sapling nutritional status, particularly by increasing leaf N concentrations in all three species, and higher soil N contents in fertilized pots further supported increased nutrient availability.

According to our hypotheses, our results show that (i) fertilization has species-specific effects on gas exchange, hydraulic traits and growth responses of studied tree saplings during drought and post-drought; (ii) although fertilization is sometimes reported to reduce the drought resistance in saplings, this was not observed in the species studied; and (iii) fertilization generally enhanced photosynthesis, stem and root hydraulic conductance, and growth under well-watered conditions, but these benefits were not consistently maintained when saplings suffered drought. Among the study species, oak kept or even enhanced its physiological and growth responses after repeated drought when fertilized, whereas the positive effects of fertilization on pine and beech diminished notably due to drought. These findings suggest that fertilization may improve sapling performance in some species, but its effectiveness under drought depends on species-specific physiology and the environmental context. Because our study was conducted on potted saplings under controlled drought conditions, the results should not be directly extrapolated to field conditions, where rooting depth, soil heterogeneity, microbial interactions and naturally fluctuating drought intensity may modify tree responses. We, however, recommend tests with several drought and fertilization intensities before use of fertilization to improve the quality of afforestation material.

## Supplementary Material

Supplementary_materials_tpag089

## Data Availability

The data that support the findings of this study are available from the corresponding author upon reasonable request.
