# Supplementary material for: Effects of fertilization on drought responses in saplings of three European trees species
Source: Tree Physiol. 2026 Jun 29;46(8):tpag089. doi: 10.1093/treephys/tpag089 (PMC13445981; doi:10.1093/treephys/tpag089)
Supplement: Supplementary file 1 [file supplementary_materials_tpag089.zip › Supplementary_materials_tpag089_Table_S2.docx]

**Table S2** Sapling height (H), basal stem diameter (D), specific leaf area (SLA), and root:shoot measured in control (C) and fertilized (F) saplings of *P. sylvestris*, *F. sylvatica* and *Q. robur* in 2023 after two previous drought cycles (summer 2021 and 2022) with three different water regimes (i.e., well-watered, WW; moderate-drought, MD; severe-drought, SD). Values represent mean ± SE. Different letters indicate significant differences among saplings of WW, MD and SD within the same fertilization group (p < 0.05), while * indicates significant differences between C and F saplings within the same water regimes (p < 0.05).

| Index | Treatment | *P. sylvestris* | | |  | *F. sylvatica* | | |  | *Q. robur* | | |
| --- | --- | --- | --- | --- | --- | --- | --- | --- | --- | --- | --- | --- |
|  |  | WW | MD | SD |  | WW | MD | SD |  | WW | MD | SD |
|  |  |  |  |  |  |  |  |  |  |  |  |  |
| H, cm | C | 93.8±2.2**^a^** | 89.3±3.7**^a^** | 81.5±3.5**^a^** |  | 68.2±2.9**^a^** | 62.0±2.4**^a^** | 58.4±6.9**^a^** |  | 78.1±3.0**^a^** | 88.6±2.5**^a^** | 81.5±5.8**^a^** |
| n=8-10 | F | 97.0±2.6**^a^** | 90.2±6.9**^ab^** | 73.0±9.4**^b^** |  | 69.5±1.4**^a^** | 59.0±3.3**^ab^** | 51.0±2.5**^b^** |  | 105.0±3.9**^a^*** | 91.2±4.7**^a^** | 91.8±6.2**^a^** |
|  |  |  |  |  |  |  |  |  |  |  |  |  |
| D, cm | C | 1.48±0.05**^a^** | 1.32±0.05**^ab^** | 1.24±0.06**^b^** |  | 1.21±0.03**^a^** | 1.13±0.05**^a^** | 1.13±0.06**^a^** |  | 0.98±0.05**^a^** | 0.98±0.04**^a^** | 0.97±0.03**^a^** |
| n=8-10 | F | 1.55±0.03**^a^** | 1.47±0.05**^a^** | 1.37±0.11**^a^** |  | 1.24±0.02**^a^** | 1.14±0.06**^ab^** | 1.07±0.01**^b^** |  | 1.13±0.07**^a^*** | 1.00±0.07**^a^** | 1.02±0.05**^a^** |
|  |  |  |  |  |  |  |  |  |  |  |  |  |
| SLA, cm^2^/g | C | 43.5±3.1^b^ | 64.1±1.9^a^ | 59.8±1.8^a^* |  | 190±10.4^b^ | 247±10.0^a^ | 209±16.9^ab^ |  | 145±9.4^a^ | 145±9.4^a^ | 140±2.5^a^ |
| n=5-8 | F | 63.7±4.4^a^* | 46.5±7.5^ab^ | 41.3±3.2^b^ |  | 284±6.4^a^* | 264±8.9^ab^ | 245±11.7^b^ |  | 173±10.7^a^ | 166±5.6^a^ | 168±4.1^a^* |
|  |  |  |  |  |  |  |  |  |  |  |  |  |
| root:shoot | C | 0.53±0.08**^a^** | 0.62±0.06**^a^** | 0.47±0.05**^a^** |  | 1.19±0.07**^a^** | 1.26±0.47**^ab^** | 2.18±0.50**^b^** |  | 2.38±0.24**^a^** | 2.48±0.47**^a^** | 1.69±0.31**^a^** |
| n=3-8 | F | 0.40±0.07**^a^** | 0.38±0.02**^a^** | 0.34±0.03**^a^** |  | 1.07±0.04**^a^** | 1.02±0.19**^a^** | 0.47±0.24**^b^*** |  | 1.98±0.04**^a^** | 2.65±0.28**^a^** | 2.56±0.54**^a^** |
|  |  |  |  |  |  |  |  |  |  |  |  |  |
