# Supplementary material for: Effects of fertilization on drought responses in saplings of three European trees species
Source: Tree Physiol. 2026 Jun 29;46(8):tpag089. doi: 10.1093/treephys/tpag089 (PMC13445981; doi:10.1093/treephys/tpag089)
Supplement: Supplementary file 1 [file supplementary_materials_tpag089.zip › Supplementary_materials_tpag089_Table_S1.docx]

**Table S1** Leaf nutrient concentrations and soil N content of control (C) and fertilized (F) saplings under well-watered conditions measured in 2023. Dried leaf tissues were analyzed for nitrogen (N), phosphorus (P), and potassium (K) concentrations, expressed as percentage of leaf dry mass. Soil N content is expressed as percentage of soil dry mass. Mean ± SE. * indicates significant differences between C and F saplings within the same species and variable (p < 0.05). Soil N data were obtained from Feng et al. (2026)

| Species | Fertilization | Leaf nutrient concentration (%) | | | Soil N content (%) |
| --- | --- | --- | --- | --- | --- |
|  |  | N | P | K |  |
| *P. sylvestris* | C | 1.31±0.03 | 0.14±0.01 | 0.73±0.04 | 0.23±0.01 |
|  | F | 1.79±0.13 * | 0.15±0.02 | 0.94±0.05 * | 0.30±0.02 |
| *F. sylvatica* | C | 1.40±0.25 | 0.22±0.01 | 0.87±0.01 | 0.26±0.01 |
|  | F | 2.29±0.13 * | 0.29±0.05 | 0.87±0.04 | 0.35±0.02 * |
| *Q. robur* | C | 1.42±0.14 | 0.24±0.03 | 0.72±0.07 | 0.24±0.01 |
|  | F | 1.94±0.02 * | 0.28±0.02 | 0.87±0.08 | 0.36±0.01 * |
