# Supplementary material for: Effects of fertilization on drought responses in saplings of three European trees species
Source: Tree Physiol. 2026 Jun 29;46(8):tpag089. doi: 10.1093/treephys/tpag089 (PMC13445981; doi:10.1093/treephys/tpag089)
Supplement: Supplementary file 1 [file supplementary_materials_tpag089.zip › Supplementary_materials_tpag089_Fig_S1.pdf]

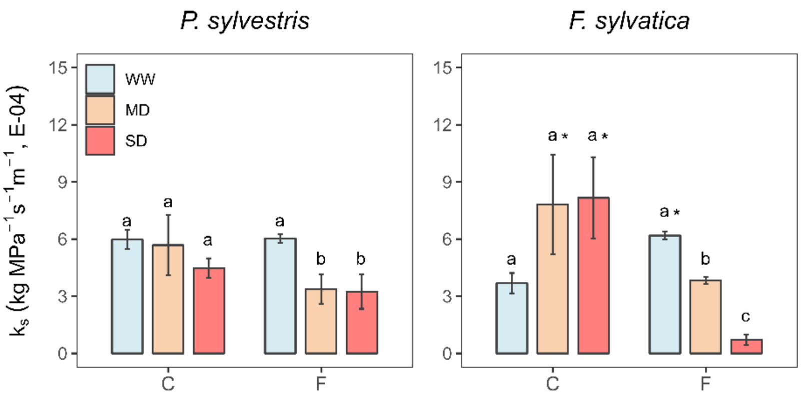

Figure S1 Specific stem conductivity (k_s) measured on control (C) and fertilized (F) saplings of *P. sylvestris* and *F. sylvatica* in 2023 after two previous drought cycles (summer 2021 and 2022) with three different water regimes (i.e., blue: well-watered, WW; orange: moderate-drought, MD; red: severe-drought, SD). Mean ± SE, n = 4-6. Different letters indicate significant differences among saplings of WW, MD and SD within the same fertilization group ($p < 0.05$), while * indicates significant differences between C and F saplings within the same water regime ($p < 0.05$). k_s measurements were not possible for *Q. robur*.
